# Awareness of Carpal Tunnel Syndrome Among the Middle-Aged Population in Riyadh, Saudi Arabia

**DOI:** 10.7759/cureus.49544

**Published:** 2023-11-28

**Authors:** Moaath A Alamir, Rakan K Alfouzan, Abdullah Alhumaidan, Hesham S Abozaid, Hamad M Alhudhaif, Rakan Alsuhaim, Mohammed A Alkholaifi

**Affiliations:** 1 Department of Surgery, Imam Mohammad Ibn Saud Islamic University, Riyadh, SAU; 2 Department of Medicine, Imam Mohammad Ibn Saud Islamic University, Riyadh, SAU; 3 Department of Anesthesia, King Faisal Specialist Hospital and Research Centre, Riyadh, SAU

**Keywords:** carpal tunnel surgery, carpal tunnel release, neuroimaging studies, cts syndrome, carpal tunnel syndome

## Abstract

Background

Carpal tunnel syndrome (CTS), a common and often underdiagnosed condition, imposes a significant burden on individuals, particularly in middle-aged populations. This study aims to assess the awareness and prevalence of CTS among middle-aged residents in Riyadh, highlighting the crucial need for targeted educational initiatives to address knowledge gaps.

Methodology

A cross-sectional study design was employed to capture a snapshot of CTS awareness and prevalence among the middle-aged population in Riyadh, Saudi Arabia. Participants aged 40-60 residing in Riyadh were included. A self-reported questionnaire gathered data on demographics, CTS diagnosis, and awareness levels regarding symptoms, risk factors, treatment, and the impact of CTS. Statistical analysis included descriptive statistics and Chi-squared tests.

Results

We collected 415 valid responses to the questionnaire. The demographic profile revealed a balanced gender distribution, with 48.4% (n=201) males and 51.6% (n=214) females, and a majority (66.5%; n=276) within the 40-50 age range. A substantial 87.2% (n=362) reported no prior CTS diagnosis. Neuroimaging studies were infrequently conducted at 15.9% (n=66). Participants exhibited significant knowledge gaps, notably in the awareness of CTS diagnosis 66.3% (n=275) uncertainty, symptoms 55.4% (n=230) lack of knowledge, and risk factors 54.7% (n=227) lack of awareness. The results indicated uncertainty regarding the relationship between CTS and diabetes 63.9% (n=265) and knowledge gaps on anesthesia methods for CTS operations 64.1% (n=294). The association between age and CTS diagnosis was significant (p-value 0.004), while awareness did not significantly vary with gender or age.

Conclusion

This study unveils a substantial lack of awareness about CTS among the middle-aged population in Riyadh, emphasizing the need for targeted educational interventions. The prevalence of CTS remains underreported, with a notable gap in understanding symptoms, risk factors, and available treatments; the findings underscore the vital importance of targeted educational programs designed to raise public awareness, bridge information gaps, and empower individuals to make informed decisions about their hand health.

## Introduction

Carpal tunnel syndrome (CTS) is a common and complex musculoskeletal condition distinguished by the constriction of the carpal tunnel-a narrow region where the median nerve is compressed [1,2]. A variety of symptoms result from this compression, including numbness and tingling, discomfort, and weakness in the hand and wrist [1,3]. Comprehending carpal tunnel syndrome (CTS) requires careful attention to the complex anatomical structure of the wrist, as any disruption to the integrity of the carpal tunnel may lead to the formation of this syndrome [4,5].

Carpal tunnel syndrome is a condition that frequently results from a confluence of physiological and occupational variables [6]. Constantly encountered in a variety of occupational settings, repetitive hand and wrist movements considerably contribute to the development of CTS [6]. Moreover, exposure to vibrations and prolonged or uncomfortable wrist positions can further intensify the potential danger [7]. Systemic disorders, like diabetes, rheumatoid arthritis, and hormonal fluctuations during pregnancy, provide further intricacies to the etiology of CTS beyond the confines of the workplace [8,9].

As CTS continues to impact a wide range of demographic groups, it becomes increasingly vital to comprehend patterns of prevalence. CTS, which was once restricted to particular occupational groups, has since spread to several sectors, reflecting the pervasive integration of technology and the increasing prevalence of sedentary lifestyles [10,11]. It is critical to investigate the demographic composition and identify populations at risk in order to develop interventions that effectively target the changing characteristics of those impacted by CTS.

An extensive variety of risk factors collectively increase an individual's vulnerability to carpal tunnel syndrome. Although occupational exposures continue to be significant, non-occupational determinants, including age, gender, obesity, and specific medical disorders, still have substantial implications. It is critical to acknowledge these elements in order to develop a comprehensive comprehension of personal risk profiles and to guide the development of focused preventative measures [12].

The severity of complications that can result from chronic or untreated carpal tunnel syndrome emphasizes the critical nature of prompt management. If left untreated, CTS can give rise to many consequences, including functional disability, diminished quality of life, and severe economic ramifications [12,13]. Prolonged instances of this condition may result in the deterioration of the muscles located at the base of the thumb, which can have a detrimental effect on the general strength and dexterity of the hand [13]. These issues underscore the necessity of adopting a comprehensive strategy in the management of CTS, which includes therapeutic therapies as well as preventative measures. Our understanding of the prevalence of CTS among the general population in the Riyadh region is limited; the purpose of this study is to determine the prevalence of CTS and the level of awareness among middle-aged individuals in Riyadh.

## Materials and methods

The study design adopted a cross-sectional design to capture a snapshot of CTS awareness among the middle-aged demographic in Riyadh. This design facilitated a comprehensive examination of participants' knowledge, considering factors such as diagnosis, symptoms, risk factors, treatment options, precautions, and the impact of CTS on their daily lives. Our sample size was 415 participants regarding the inclusion and exclusion criteria; inclusion criteria encompassed participants residing in the city of Riyadh within the age bracket of 40 to 60 years. Adults within this age range were considered suitable candidates for the study. Exclusion criteria involved individuals residing outside the geographical boundaries of Riyadh, pediatric participants, and the elderly, acknowledging that the study's focus was on the middle-aged population.

Prior to participation, explicit consent was obtained from each individual, emphasizing their voluntary involvement in the study. Ethical approval was sought and granted by the relevant institutional review board to ensure the study adhered to ethical standards and protected participants' rights. The research project was authorized by the ethical research committee of the Institutional Review Board (IRB) Registration of Imam Mohammad Ibn Saud Islamic University (IMSIU), Riyadh, Saudi Arabia. 

The research data were collected through an online self-reported questionnaire, which was adopted from a previously validated questionnaire from an Al-Majmaah study [14], that were collected over a span of two months, which were designed to gather demographic information, including gender and age. Participants were also asked if they had been previously diagnosed with CTS and had had a neuroimaging study done for them.

The questionnaire delved into aspects of awareness, assessing participants' knowledge regarding CTS diagnosis, symptoms, risk factors, treatment options, precautions, and the perceived impact of CTS on their daily lives. Lastly, the respondents were asked if they think there is a relationship between Diabetes mellitus and CTS and what type of anesthesia is used for carpal tunnel surgery.

The statistical analysis was done on Microsoft Excel (Microsoft, Redmond, Washington), and it was used for data entry, cleaning, and coding, while SPSS version 26 (IBM Inc., Armonk, New York) was used for data analysis. The collected data underwent statistical analysis using appropriate measures, such as descriptive statistics, to summarize demographic characteristics and awareness levels. Comparative analyses were conducted to identify patterns and differences in awareness across demographic groups, including the t-test and Chi-squared test. All statements with a p-value lower than 0.05 are considered significant.

## Results

We collected 415 valid responses to the questionnaire. The demographic profile of the participants revealed a balanced gender distribution, with 48.4% (n=201) males and 51.6% (n=214) females. In terms of age, the majority fell within the 40-50 age range, constituting 66.5% (n=276), while 33.5% (n=139) belonged to the 51-60 age bracket. Regarding the prevalence of carpal tunnel syndrome (CTS), 87.2% (n=362) of participants reported no previous diagnosis, while 12.8% (n=53) confirmed a positive diagnosis. Neuroimaging studies were not commonly conducted, with 84.1% (n=349) reporting no such studies and 15.9% (n=66) having undergone neuroimaging for CTS (Table 1).

**Table 1:** Demographic factors of the participants and prevalence of CTS (N=415) CTS - carpal tunnel syndrome

Demographics		Count	%
Gender	Male	201	48.4%
	Female	214	51.6%
Age	40-50	276	66.5%
	51-60	139	33.5%
Have you been diagnosed with CTS?	No	362	87.2%
	Yes	53	12.8%
Have you had a neuroimaging study done for you?	No	349	84.1%
	Yes	66	15.9%

Across all categories, a significant proportion of respondents indicated a lack of knowledge. Notably, the highest rate of uncertainty was observed in the awareness about the diagnosis of CTS, with 66.3% (n=275) reporting "I do not know." For the diagnosis of CTS, only 24.1% (n=100) of participants were aware that neurography studies could be utilized for diagnosis, 17.3% (n=72) recognized the significance of medical history, and 22.4% (n=93) acknowledged clinical diagnosis as a diagnostic method. Regarding awareness of CTS symptoms, 31.1% (n=129) identified wrist pain, 25.3% (n=105) recognized tingling and numbness in specific fingers, 11.3% (n=47) associated CTS with muscle wasting, and 23.4% (n=97) identified weakness in hand grip. However, a substantial proportion of 55.4% (n=230) reported a lack of knowledge regarding CTS symptoms. In terms of risk factors, varying levels of awareness were observed. Diabetic mellitus was acknowledged by 21.2% (n=88), dysthyroid by 10.4% (n=43), obesity by 10.8% (n=45), gender by 7.2% (n=30), frequent movements by 16.9% (n=70), and rheumatoid arthritis by 28.9% (n=120). Nevertheless, 54.7% (n=227) indicated a lack of awareness regarding CTS risk factors. Concerning awareness of CTS treatment options, 15.4% (n=64) recognized nonsteroidal anti-inflammatory drugs, 22.2% (n=92) identified supportive ligaments, 22.4% (n=93) acknowledged release surgery, and 23.9% (n=99) recognized cortisone injections as potential interventions for CTS. A notable 62.7% (n=260) reported a lack of knowledge about CTS treatment options. For precautions to avoid CTS, 28.7% (n=119) recognized avoiding repetitive wrist movement, 19.8% (n=82) identified keeping the wrist straight while sitting, and an additional 19.8% (n=82) acknowledged staying warm and avoiding cold environments. However, 59.5% (n=247) expressed a lack of awareness regarding CTS precautions. Lastly, regarding the effects of CTS on patients, 25.1% (n=104) acknowledged its effects on sleep, 34.7% (n=144) recognized its influence on daily and social life, and 27.7% (n=115) identified its impact on overall function. A substantial 53.3% (n=221) indicated a lack of knowledge about the effects of CTS on patients (Table 2).

**Table 2:** The awareness of the participants toward different aspects of CTS CTS - carpal tunnel syndrome

Awareness		Count	%
Awareness about the diagnosis of CTS	Neurography study	100	24.1%
	Medical history	72	17.3%
	Clinical diagnosis	93	22.4%
	I do not know	275	66.3%
Awareness about symptoms of CTS	Wrist pain	129	31.1%
	Tingling and numbness in the thumb, index finger, and middle finger	105	25.3%
	Muscle wasting	47	11.3%
	Weakness in hand grip	97	23.4%
	I do not know	230	55.4%
Awareness about risk factors of CTS	Diabetic mellitus	88	21.2%
	Dysthyroid	43	10.4%
	Obesity	45	10.8%
	Gender	30	7.2%
	Frequent movements	70	16.9%
	Rheumatoid arthritis	120	28.9%
	I do not know	227	54.7%
Awareness about the treatment of CTS	Nonsteroidal anti-inflammatory drugs	64	15.4%
	Supportive ligament	92	22.2%
	Release surgery for carpal tunnel syndrome	93	22.4%
	Cortisone injections	99	23.9%
	I do not know	260	62.7%
Awareness about precautions to avoid CTS	Avoid repetitive wrist movement	119	28.7%
	Keep the wrist straight while sitting	82	19.8%
	Stay warm and stay out of the cold	82	19.8%
	I do not know	247	59.5%
Effects of CTS on the patients	Affects the patient's sleep	104	25.1%
	It affects the patient's daily and social life	144	34.7%
	Affects the patient's function	115	27.7%
	I do not know	221	53.3%

**Figure 1:**
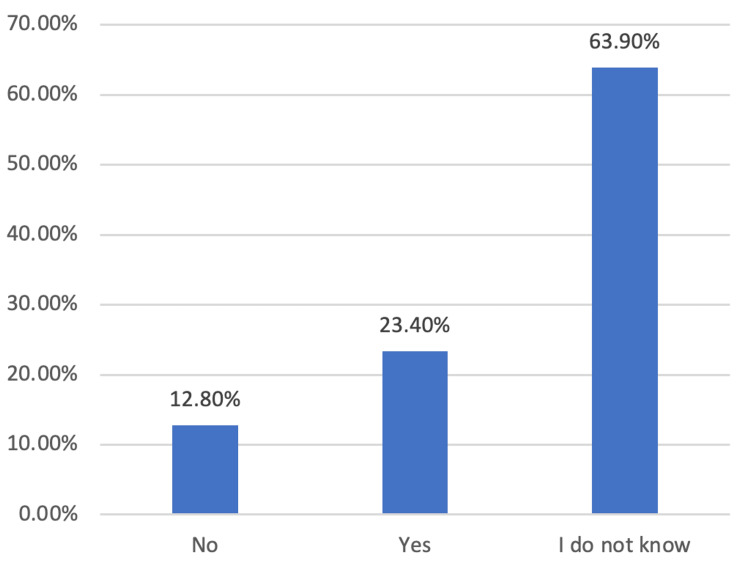
When asked about the relationship of diabetes to carpal tunnel syndrome in the questionnaire, the results were as follows Y-axis: percentage of the population

**Figure 2:**
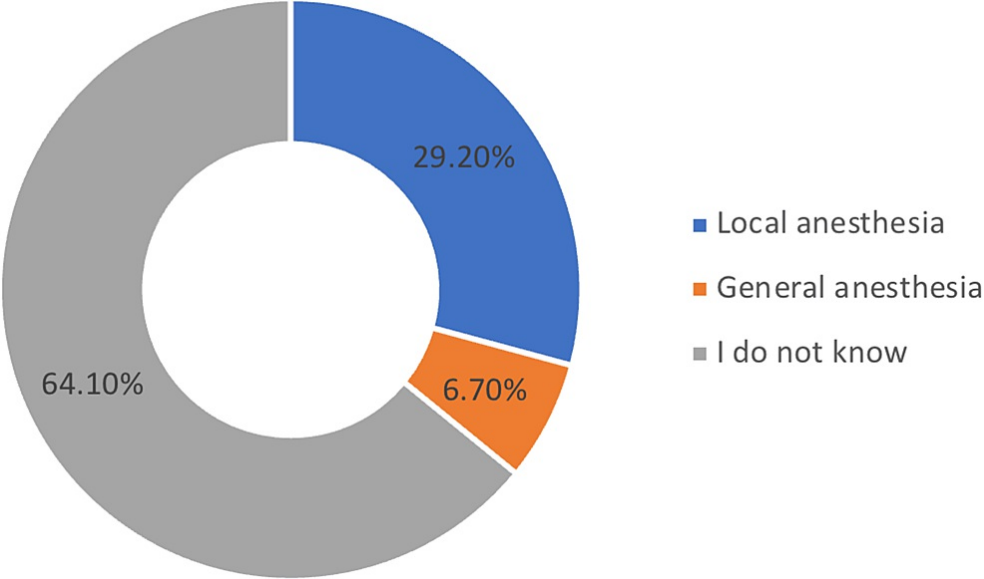
When asked about which type of anesthesia is used for carpal tunnel syndrome operations in the questionnaire, the results were as follows

In Figure 1, participants were queried about their perception of the relationship between carpal tunnel syndrome (CTS) and diabetes. The results indicated that 23.4% (n=97) affirmed a belief in such a connection, while 12.8% (n=53) negated any association. Notably, a substantial majority, 63.9% (n=265), expressed uncertainty regarding the potential link between CTS and diabetes. While in Figure 2, participants were asked about their knowledge of anesthesia methods used in CTS operations. Among the respondents, 29.2% (n=121) identified local anesthesia, 6.7% (n=28) recognized general anesthesia, and the majority, 64.1% (n=266), conveyed a lack of knowledge regarding the anesthesia methods employed in CTS surgeries.

Table 3 explored the relationship between the diagnosis of carpal tunnel syndrome (CTS) and demographic factors. The results indicated that gender did not significantly influence the likelihood of CTS diagnosis, with 87.1% (n=175) of males and 87.4% (n=187) of females reporting no diagnosis. However, age exhibited a notable association, as 90.6% (n=250) of individuals aged 40-50 reported no diagnosis compared to 80.6% (n=112) in the 51-60 age group. The difference in diagnosis rates between these age groups was statistically significant (p-value 0.004).

**Table 3:** The relation between CTS and demographic factors (N=415) CTS - carpal tunnel syndrome

Demographics		Had you been diagnosed with CTS?				
		No		Yes		p-value
		Count	%	Count	%	
Gender	Male	175	87.1%	26	12.9%	0.923
	Female	187	87.4%	27	12.6%	
Age	40-50	250	90.6%	26	9.4%	0.004*
	51-60	112	80.6%	27	19.4%	

Table 4 delved into the association between the level of awareness toward carpal tunnel syndrome (CTS) and demographic factors. Gender did not reveal a significant association with awareness levels, as 86.1% (n=173) of males and 89.7% (n=192) of females demonstrated inadequate awareness. Similarly, age did not exhibit a notable association, with 88.8% (n=245) of those aged 40-50 and 86.3% (n=120) of those aged 51-60 showing inadequate awareness. Regarding a previous diagnosis of CTS, there was a trend towards higher awareness among those who had been diagnosed, with 81.1% (n=43) demonstrating inadequate awareness compared to 89.0% (n=322) of those who had not been diagnosed. However, the association did not reach statistical significance (p-value 0.102).

**Table 4:** The association between level of awareness and demographic factors (N=415) CTS: Carpal Tunnel Syndrome

Demographics		Awareness toward CTS				
		Inadequate		Adequate		P-value
		Count	N%	Count	N%	
Gender	Male	173	86.1%	28	13.9%	0.254
	Female	192	89.7%	22	10.3%	
Age	40-50	245	88.8%	31	11.2%	0.472
	51-60	120	86.3%	19	13.7%	
Had you been diagnosed with CTS?	No	322	89.0%	40	11.0%	0.102
	Yes	43	81.1%	10	18.9%	

## Discussion

Carpal tunnel syndrome (CTS) remains a pervasive and sometimes misdiagnosed disorder that significantly affects individuals, with a particular burden on those in the middle-aged demographic [15,16]. Diverse prevalence rates have been documented in studies; Mondelli et al. observed an incidence of 5.8 per 1000 person-years among the general population [17].

Nonetheless, the actual prevalence can be greater due to the fact that CTS is frequently overlooked, which contributes to the underestimation of its effects. The present investigation revealed a prevalence rate of 12% for CTS. This prevalence is more than that which was reported in other Saudi Arabian studies, notably a Western Region research [18] and the Al-Majmaah study [14], which had prevalence rates of 2% and 14%, respectively. In contrast, a study conducted among the general populace of Riyadh, Saudi Arabia, observed that the prevalence of CTS symptoms was 50% when the overall prevalence of warning symptoms was also considered [19].

The correlation between age and the diagnosis of CTS is consistent with long-standing patterns since the incidence of CTS generally rises with advancing age [20-22]. Nevertheless, the absence of a substantial gender impact on the diagnosis of CTS is intriguing, given that females frequently have a greater prevalence [23-25]. Additional research is required to identify occupational and lifestyle factors that may contribute to the development of CTS in individuals of both sexes, as shown by this variation. According to the results of our research, the surveyed population lacks sufficient understanding of CTS, specifically concerning its treatment, effects, and preventative measures. This supports the results of a previous study done in the Western Region of Saudi Arabia [18] and the Al-Jouf region study [26], which found that the majority of participants lacked awareness. In contrast, our results contradict those of research conducted in the Saudi Arabian city of Al-Majmaah, which concluded that the adult population possessed an adequate level of awareness [14]. The considerable degree of ambiguity among participants about the diagnosis of CTS is consistent with results reported in previous research, underscoring the criticality of heightened educational initiatives [27]. In particular, it is regrettable that neurography investigations are not more widely recognized as a diagnostic tool, despite the fact that the scientific literature attests to their effectiveness in identifying CTS [28]. The evident dearth of understanding pertaining to symptoms, risk factors, and treatment alternatives underscores the urgent requirement for public health campaigns and focused educational efforts. An investigation conducted by Dale et al. revealed that individualized educational initiatives substantially enhanced patients' understanding of CTS and competence in self-management [29].

A substantial majority, 63.9% (n=265), has shown ambiguity over the potential correlation between CTS and diabetes, which highlights an area of knowledge that necessitates further investigation.

While several studies indicate a potential association between CTS and diabetes, others posit a more intricate connection that is also impacted by metabolic syndrome and obesity [30]. It is essential that this knowledge gap be closed in order to facilitate early detection and management for patients and healthcare practitioners alike.

The absence of substantial correlations between levels of awareness and gender or age suggests that disparities in awareness are present across all demographic categories. These findings are comparable to those of the Western area study, which found that although the difference was not statistically significant, females had marginally greater awareness than males across all factors [18]. Age was found to be substantially correlated with awareness in the Al-Jouf region study [26]. Specifically, adults between the ages of 18 and 30 had the highest level of awareness compared to other age groups. Nevertheless, the upward trajectory in consciousness observed among persons who had been previously diagnosed with CTS implies that heightened awareness may be motivated by personal encounters with the disorder. This highlights the potential ramifications of tailored educational interventions for those who do not have a preexisting diagnosis.

Limitations

Although our research offers interesting insights, it is important to acknowledge numerous limitations. The cross-sectional form of the study and its dependence on self-reported data both present the possibility of recollection bias and restrict the ability to demonstrate causation. Further investigation may benefit from the use of longitudinal methodologies and objective metrics in order to bolster the validity of the results.

## Conclusions

This research provides insights into the areas of awareness and knowledge deficiencies pertaining to carpal tunnel syndrome among the middle-aged demographic residing in Riyadh. The results emphasize the critical need for focused educational initiatives that aim to increase public consciousness, close gaps in information, and enable individuals to make well-informed choices regarding their hand health. Subsequent endeavors must prioritize comprehensive approaches, encompassing collaboration with healthcare practitioners, community engagement, and digital health tools, in order to effectively tackle the various obstacles presented by carpal tunnel syndrome within this particular demographic.
